# 7-Day Single-Lead Electrocardiogram Monitoring for Atrial Fibrillation Detection in Routine Health Screenings

**DOI:** 10.1016/j.jacasi.2026.02.024

**Published:** 2026-05-05

**Authors:** Muhan Yeo, So-Ryoung Lee, Jin Ho Park, Seo Eun Hwang, Hyuktae Kwon, Heesun Lee, Tae-Min Rhee, Bumjo Oh, Young-Sang Kim, Jae Kyung Choi, Jinyoung Shin, Hyejin Chun, Hyo-Jeong Ahn, Soonil Kwon, Seil Oh, Su-Yeon Choi, Eue-Keun Choi

**Affiliations:** a Department of Internal Medicine, College of Medicine, Seoul National University, Seoul, South Korea; b Department of Internal Medicine, Seoul National University Hospital, Seoul, South Korea; c Department of Family Medicine, Seoul National University Hospital, College of Medicine, Seoul National University, Seoul, South Korea; d Department of Internal Medicine, Gangnam Center, Seoul National University Hospital Healthcare System, Seoul, South Korea; e Department of Family Medicine and Healthcare Center, SMG-SNU Boramae Medical Center, Seoul, South Korea; f Department of Family Medicine, CHA University; CHA Global Clinical Trials Center; Global Clinical Research Center, CHA Future Medicine Research Institute, CHA University, Pocheon, South Korea; g Department of Family Medicine, Konkuk University Medical Center, Research Institute of Medical Science, School of Medicine, Konkuk University, Seoul, South Korea; h Department of Family Medicine, Ewha Womans University Seoul Hospital, College of Medicine, Ewha Womans University, Seoul, South Korea; i Department of Internal Medicine, SMG-SNU Boramae Medical Center, Seoul, South Korea

**Keywords:** ambulatory, artificial intelligence, atrial fibrillation, electrocardiography, mass screening, stroke

Atrial fibrillation (AF) is common, often silent, and linked to stroke and adverse outcomes.^1^^,^^2^ Opportunistic screening is recommended for individuals at elevated risk of stroke, but detection during routine health checkups is limited when relying on a single 12-lead electrocardiogram (ECG).^3^^,^^4^ Prolonged ambulatory monitoring increases diagnostic yield, and adhesive single-lead patches offer a scalable option for short-term continuous monitoring with automated interpretation.^5^, ^6^, ^7^, ^8^ In South Korea, the national health screening participation rate exceeds 75%,^9^ providing a unique infrastructure for evaluating the feasibility of large-scale AF detection. We evaluated whether 7-day single-lead patch monitoring increases AF detection in real-world health screenings and characterized AF burden, time to first detection, and simple clinical predictors to guide targeted deployment.

The AF-CARE (Atrial Fibrillation Screening With mobiCARE in Subjects With Health Care Examination; NCT06036342) study is a prospective, multicenter cohort at 6 Korean screening centers (April 2022 to December 2023). Adults aged ≥19 years without known AF presenting for national or voluntary health checkups were eligible if their CHA_2_DS_2_-VASc score was ≥2. Exclusions included implanted pacemakers, skin conditions precluding patch use, or life-threatening arrhythmias. The protocol was approved by institutional review boards at all sites (Seoul National University Hospital IRB H-2014-226-1217), and all participants provided written informed consent.

Participants wore a single-lead adhesive ECG patch (mobiCARE MC200M; Seers Technology) for up to 7 days. Device algorithms by semantic segmentation flagged AF, and 3 cardiologists adjudicated. A 12-lead ECG performed during the checkup was used for comparison. The primary endpoint was AF detection (defined as ≥30 seconds) with patch monitoring versus single-timepoint 12-lead ECG. We summarized the AF burden (percentage of time in AF), longest episode duration, and cumulative detection by day. Multivariable logistic regression identified the independent predictors of AF detection; variables with >25% missingness (years of education, monthly income, serum total CO_2_) were excluded, and the remaining missing values were handled using MICE (multivariate imputation by chained equations; m = 5, pooled using Rubin’s rules).

We enrolled 2,978 participants of mean age: 66 ± 8 years; 1,161 were male (39%), with a median CHA_2_DS_2_-VASc score of 2 (Q1-Q3: 2-3). A 12-lead ECG reading was available for 2,556 participants (86%). AF ≥30s was detected in 44 of 2,978 (1.48%; 95% CI: 1.08%-1.98%) by the 7-day patch versus 6 of 2,556 (0.23%; 95% CI: 0.09%-0.51%) by single time point 12-lead ECG, yielding a 6.29-fold higher detection (relative risk [RR]; 95% CI: 2.69-14.75) and excess detection 1.24% (absolute risk difference; 95% CI: 0.77-1.71) (Figure 1A). The benefit was observed in both sexes: RR: 6.57 (95% CI: 2.32-18.6) in men and 5.92 (95% CI: 1.35-26.0) in women. By age, the RR appeared higher in older groups: 1.54 (95% CI: 0.45-5.24) for 19-64 years, 12.2 (95% CI: 2.91-51.2) for 65-74 years, and 14.3 (95% CI: 0.84-245) for ≥75 years.

**Figure 1:**
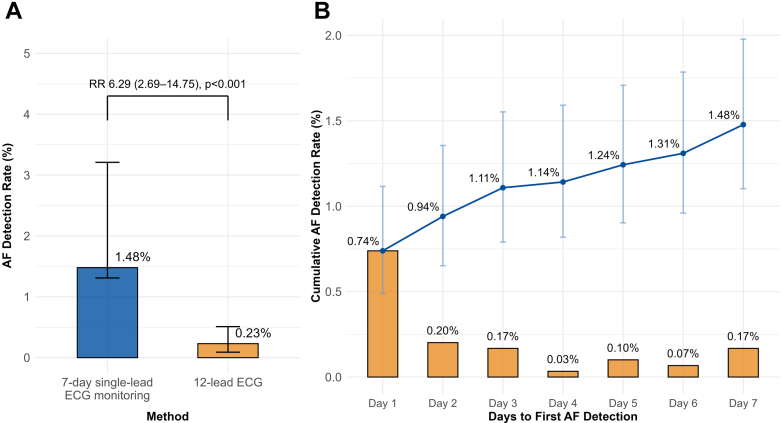
Atrial Fibrillation Detection With 7-Day Single-Lead Electrocardiographic Monitoring (A) Atrial fibrillation detection rate with 7-day single-lead ECG patch monitoring versus single-timepoint 12-lead ECG among eligible participants. Bars indicate detection rates, and error bars indicate 95% confidence intervals; the relative risk is shown above the comparison. (B) Time to first atrial fibrillation detection during patch monitoring. Bars show the daily proportion of newly detected atrial fibrillation cases (incident detections by day), and points/line show the cumulative atrial fibrillation detection proportion over 7 days. Error bars indicate 95% CI for the cumulative detection proportion. Half of atrial fibrillation cases were identified on day 1 and 75% by day 3. AF = atrial fibrillation; ECG = electrocardiogram; RR = relative risk.

To address potential bias from incomplete 12-lead ECG availability (86%), we performed inverse-probability weighting. Propensity scores for 12-lead ECG were estimated using multivariable logistic regression including age, sex, CHA_2_DS_2_-VASc score, body mass index, hypertension, diabetes mellitus, ischemic heart disease (IHD), hyperlipidemia, chronic kidney disease, previous stroke, family history of IHD/stroke, and oral anticoagulant or aspirin use. The weighted absolute risk difference and RR of AF detection were 1.53% (95% CI: 0.46%-2.56%) and 8.23 (95% CI: 2.99-22.7), respectively.

Among the 44 AF-positive participants, the median AF burden was 4.1% (Q1-Q3: 0.4-11.9) and median longest episode was 3.0 hours (Q1-Q3: 0.3-11.6). Fifty percent (22 of 44) of AF was identified on day 1, and 75% (33 of 44) was identified by monitoring to day 3 (Figure 1B). Symptoms were reported by 31.8% (14 of 44) of the AF-positive participants, most commonly palpitations.

In multivariable analysis adjusted for age, sex, hyperlipidemia, exercise category, family history of IHD, hemoglobin, platelet count, triglycerides, serum calcium, left ventricular hypertrophy (LVH) and atrial premature beats (APB) in 12-lead ECG, and proteinuria category, older age, LVH and APB on 12-lead ECG, and family history of IHD independently predicted AF detection (all *P* < 0.05).

Among the 2,717 participants who completed the tolerability questionnaire, 60% (1,628 of 2,717) reported they could wear the patch ≥5 days; skin irritation was the most frequent complaint (51%; 1,395 of 2,717), and 7.9% (214 of 2,717) discontinued early due to discomfort. The median total wear time in the overall cohort was 5.9 days (Q1-Q3: 4.0-6.6) and longer wear correlated with higher detection.

These findings suggest that, in health screening populations selected by CHA_2_DS_2_-VASc score, short-term continuous patch monitoring substantially increases AF detection compared with a single 12-lead ECG. Detected AF frequently exhibited clinically meaningful burden and multihour episodes, supporting a potential downstream impact on stroke prevention and rhythm management. The front-loaded yield (half detected on day 1 and three-quarters by day 3) suggests that 3 to 7 days of monitoring may balance detection efficiency with user comfort and program cost. The number needed to screen was 90 for 3 days and 68 for 7 days, and stepwise strategies (eg, extending monitoring from 3-4 to 7 days if AF is not detected) may offer a practical compromise. Simple features available at the time of screening (age and 12-lead ECG findings such as LVH and APB) could help target patch deployment to those with the highest probability of detection, improving the efficiency of screening programs.

This real-world cohort complements prior studies of patch-based screening by demonstrating scalability within routine health checkups. Although prior trials established the diagnostic advantage of prolonged monitoring, our study provides implementation evidence from an Asian, risk-enriched health screening population for integrating short-term patch monitoring into real-world care. Although our comparison cannot establish clinical outcome benefits from earlier AF identification, the observed duration and burden fall within ranges linked to elevated thromboembolic risk, underscoring the clinical relevance. Limitations include selection bias inherent to health checkup cohorts, limited ethnic diversity, and reliance on a single device and algorithm.

In summary, continuous 7-day single-lead ECG patch monitoring improved AF detection 6-fold versus a single 12-lead ECG among high-risk adults undergoing routine screenings. The combination of meaningful AF burden, acceptable tolerability, and actionable clinical predictors supports integrating short-term patch monitoring into AF screening protocols in health screening settings.

## Funding Support and Author Disclosures

Supported by PACEN (MOHW; RS-2021-KH119931), NRF (MSIT; IRIS RS-2024-00340590), and Daewoong Pharmaceutical. Dr E-K Choi has received grants or fees from Abbott, Bayer, BMS/Pfizer, Biosense Webster, Chong Kun Dang, Daewoong, Daiichi-Sankyo, DeepQure, Dreamtech, Jeil, Medtronic, Samjinpharm, Samsung Electronics, Seers, and Skylabs. Dr S-Y Choi has received grants or fees from Daewoong. All other authors have reported that they have no relationships relevant to the contents of this paper to disclose.
